# Scalable Fabrication of Natural-Fiber Reinforced Composites with Electromagnetic Interference Shielding Properties by Incorporating Powdered Activated Carbon

**DOI:** 10.3390/ma9010010

**Published:** 2015-12-25

**Authors:** Changlei Xia, Shifeng Zhang, Han Ren, Sheldon Q. Shi, Hualiang Zhang, Liping Cai, Jianzhang Li

**Affiliations:** 1 MOE Key Laboratory of Wooden Material Science and Application, Beijing Key Laboratory of Wood Science and Engineering, Beijing Forestry University, Beijing 100083, China; lijianzhang126@126.com; 2 Department of Mechanical and Energy Engineering, University of North Texas, Denton, TX 76203, USA; changlei.xia@unt.edu (C.X.); liping.cai@unt.edu (L.C.); 3 Department of Electrical Engineering, University of North Texas, Denton, TX 76203, USA; hanren@my.unt.edu (H.R.); hualiang.zhang@unt.edu (H.Z.)

**Keywords:** natural-fiber, composites, electromagnetic interference (EMI) shielding, activated carbon, vacuum-assisted resin transfer molding (VARTM)

## Abstract

Kenaf fiber—polyester composites incorporated with powdered activated carbon (PAC) were prepared using the vacuum-assisted resin transfer molding (VARTM) process. The product demonstrates the electromagnetic interference (EMI) shielding function. The kenaf fibers were retted in a pressured reactor to remove the lignin and extractives in the fiber. The PAC was loaded into the freshly retted fibers in water. The PAC loading effectiveness was determined using the Brunauer-Emmett-Teller (BET) specific surface area analysis. A higher BET value was obtained with a higher PAC loading. The transmission energies of the composites were measured by exposing the samples to the irradiation of electromagnetic waves with a variable frequency from 8 GHz to 12 GHz. As the PAC content increased from 0% to 10.0%, 20.5% and 28.9%, the EMI shielding effectiveness increased from 41.4% to 76.0%, 87.9% and 93.0%, respectively. Additionally, the EMI absorption increased from 21.2% to 31.7%, 44.7% and 64.0%, respectively. The ratio of EMI absorption/shielding of the composite at 28.9% of PAC loading was increased significantly by 37.1% as compared with the control sample. It was indicated that the incorporation of PAC into the composites was very effective for absorbing electromagnetic waves, which resulted in a decrease in secondary electromagnetic pollution.

## 1. Introduction

Electromagnetic interferences (EMI), which are conducting and radiating electromagnetic signals emitted by electrical circuits, perturb proper operation of surrounding electrical equipment or cause radiative damage to living organisms [1,2]. An abundance of EMI shielding materials and technologies have been synthesized and designed to reduce the interferences caused by electromagnetic signals, e.g., metal sheets [3], carbon materials [4,5,6,7,8,9,10,11,12,13], electroless plating [14], honeycomb design [1], and coating [15]. Three mechanisms of EMI shielding were investigated [16]: reflection, which involves the application of metal sheets; absorption mechanism, which happens in amorphous materials; and multiple reflections, which refer to the reflections at various surfaces or interfaces in the shield.

Currently, the most common EMI shielding is metal sheets (such as nickel film [17], copper film [18], and iron and cobalt particles [19]) because of their excellent reflection of electromagnetic signals. However, metal sheets also have disadvantages such as high density, corrosive action, uneconomic processing and secondary electromagnetic pollution resulting from the reflection [20]. These drawbacks deter metal sheets from practical applications in the EMI shielding field. Therefore, a need exists to develop new materials that feature similar EMI shielding characteristics as metal sheets but are easier to be manufactured, as well as more economical and portable.

Interest in the development of carbon-based EMI shielding has arisen driven because of the following advantages the material offers: corrosion-resistance, low density, environmentally friendly, and easy to manufacture. The reported EMI shielding applications of carbon materials included carbon black [10], carbon nanotube [6,9], carbon fiber [7,8], carbon nonfiber [12], activated carbon fiber [11], and graphene sheets [4,5]. Like other carbon materials, activated carbon demonstrates good EMI shielding effectiveness and EMI absorption [4,5,6,7,8,9,10,11,12]. Activated carbon is an inexpensive resource with low density, and the cost effectiveness and large production of activated carbon increase the possibility of wide utilization as an EMI shielding candidate. However, no report was found in the literature review about the use of powdered activated carbon (PAC) for EMI shielding composites. Vacuum-assisted resin transfer molding (VARTM) process was performed to manufacture PAC incorporated kenaf-fiber reinforced composites, which is turned out to be an excellent process for fabricating hybrid polymer-matrix composites [21,22,23].

Activated carbon is a crude form of graphite with a random or amorphous structure, which is highly porous with large internal surface area [24]. Activated carbon is a cheap resource with low density and electromagnetic absorption properties. The low price and large production of activated carbon increase the possibility of the wide utilization as an EMI shielding candidate. This work was aimed at developing EMI shielding composites using kenaf fibers by incorporating PAC.

## 2. Materials and Methods

### 2.1. Materials

The kenaf bast fibers were obtained from Kengro Corporation (Charleston, MS, USA). The sodium hydroxide (NaOH) solution (5%, w/v) was prepared using NaOH beads (≥97%, Acros Organics, Morris Plains, NJ, USA) and deionized water. The activated carbon (12 × 40 mesh) was purchased from Calgon Carbon Corporation (Pittsburgh, PA, USA). The unsaturated polyester AROPOL Q6585 (30% styrene, Ashland Chemicals, Roseland, NJ, USA) and tert-butyl peroxybenzoate (t-BP, 98%, Acros Organics, Morris Plains, NJ, USA) were used to fabricate the kenaf fiber reinforced composites.

### 2.2. Preparation of Powdered Activated Carbon

Using the ultra-fine pulverizing machine (RT-UF26, Rong Tsong Precision Technology Co., Taichung, Taiwan), the activated carbon was reduced to powder. According to the requirements of the American National Standards Institute/American Water Works Association (ANSI/AWWA) B600—10 standard, the particle-size distribution of PAC was: not less than 99% of the activated carbon shall pass a No. 100 sieve, not less than 95% shall pass a No. 200 sieve, and not less than 90% shall pass a No. 325 sieve. The particle-size distribution of the PAC was measured by a Beckman Coulter Delsa™ Nano C Particle Analyzer (Beckman Coulter, Inc., Irving, TX, USA). PAC was dispersed into the DI-water with a concentration of 1 mg·mL^−1^. Prior to the particle-size measurement, PAC/aqueous dispersion was treated by a VCX 1500 ultrasonic (Sonics & Materials Inc., Newtown, CT, USA) for 5 min.

### 2.3. Preparation of Preformed Mats

Measured by a Mettler-Toledo HB43-S Moisture Analyzer (Mettler-Toledo LLC, Columbus, OH, USA), the average moisture content of the kenaf bast fibers was 9.1%. A mixture of 100 g kenaf fibers and 1.8 L NaOH solution was added into a hermetical reactor (251 M, Parr Instrument Co., Moline, IL, USA). With a saturated vapor pressure of 0.60 MPa, the alkali retting process was conducted at 160 °C for one hour while the mixture was being mechanically stirred. The excessive ionic solution was removed from the kenaf fibers by gravity after cooling to room temperature, and then by hand-squeezing. After the retting process, the retted fibers were measured as 36.6% ± 1.2% of the original ones.

PAC was stirred in 1 L water at 70 °C for approximate 30 min, and then mixed with fresh alkali retted kenaf fibers with another 30 min of stirring. The mixture was formed into a preform mat with a dimension of approximate 100 × 165 × 10 mm^3^, and was dried at 105 °C for 24 h. Three different amounts (10, 20, and 30 g) of PAC (6.1% moisture content, measured by a Mettler-Toledo HB43-S Moisture Analyzer) were loaded to kenaf fibers to create three types of composites, Fiber/PAC10, Fiber/PAC20, and Fiber/PAC30.

### 2.4. Composites Fabrication through VARTM Process

Four types of composites, *i.e.*, Fiber/PAC10/polyester, Fiber/PAC20/polyester, Fiber/PAC30/polyester and Fiber/polyester (as control panels using un-treated fibers), were fabricated using the unsaturated polyester resin with 1.5% of t-BP catalyst and the VARTM process, which was described in our previous reports [25]. Briefly, after applying a mold-release agent on the surface of the mold, the preform was placed on the mold. A vacuum bag was placed over the mold. After vacuum tubes were inserted in the bag, resin infusion was carried out by a vacuum that was created between the mold and the bag. As a result, the catalyzed resin was supplied to the infusion tubes. The vacuum pulled the resin along the distribution layer into the preformed mats. A vacuum of 1.3–1.6 KPa was applied to the infusion system by the vacuum pump (Vacmobile 20/2 System with Becker U4.20, Vacmobiles.com Limited, Auckland, New Zealand). The resin was cured in the hot press with a pressure of 13 MPa. The resin-infused preforms were cured in two temperature steps, *i.e.*, 100 °C for 2 h, and then 150 °C for 2 h. Once the resin cured and cooled down to room temperature, the vacuum bag and distribution layer were removed. Before the mechanical property measurements, all the specimens were conditioned approximate 30 days to a constant weight in a conditioning chamber maintained at a relative humidity of 50% ± 2% and a temperature of 20 ± 3 °C.

### 2.5. Specific Surface Area and Pore Structure Analysis

The specific surface areas of the samples were analyzed by the surface area analyzer (3Flex, Micromeritics Instrument Corp., Norcross, GA, USA) in terms of nitrogen adsorption/desorption at 77 K. Based on the isothermal plots from *P*/*P*_0_ = 0.05 to 0.3, the surface areas of the samples were obtained in accordance with the Brunauer-Emmett-Teller (BET) model. The specific pore volume distribution of the samples was analyzed by means of the Density Functional Theory (DFT) model. 

### 2.6. Microtopography Analysis of Fibers

The surface topologies of the un-treated kenaf fibers and Fiber/PAC30 were examined using a Quanta 200 environmental scanning electron microscope (SEM, FEI Company, Hillsboro, OR, USA) with an accelerating voltage of 20 kV and magnifications of 1000× and 2000×. Prior to the SEM tests, the specimens were coated by a gold sputtering coater for 5 min to prevent charging of the specimen by the SEM electron beam.

### 2.7. Electromagnetic Interference Shielding Tests

The EMI shielding effectiveness measurement with both amplitude and phase properties were carried out using the vector network analyzer (HP E8363B, Agilent Technologies, Inc., Santa Clara, CA, USA) with a frequency ranging from 8 GHz to 12 GHz. As the most common type of network analyzer, the vector network analyzer could be also called a gain-phase meter or an automatic network analyzer. Two *S*-parameters (%), including *S*11 (reflected), and *S*21 (transmitted), were detected and recorded. The EMI shielding effectiveness (%) and EMI absorption of composites were plotted using with Equations (1) and (2), respectively.

(1) EMI shielding effectiveness (%)=(1−S21)×100

(2) EMI absorption (%)=(1−S21−S11)×100

### 2.8. Mechanical Property Tests

From each composite, twelve specimens measuring 25 × 160 × 3 mm^3^ were cut in order to examine the modulus of elasticity (MOE) and modulus of rupture (MOR) of the composites. The Shimadzu AGS-X universal testing machine was used for the examinations in accordance with the procedure of 3-point bending test described in ASTM D790 standard. Three-point bending set-up was used with a span of 50 mm and a crosshead speed of 1.3 mm/min.

### 2.9. Dynamic Mechanical Analysis

The dynamic mechanical analysis (DMA) was performed using a TA instruments Q800 DMA tester (TA instruments Inc., New Castle, DE, USA). Each specimen measuring about 1 × 4 × 30 mm^3^ were used for the tests. Three-point bending with a gauge length of 25.4 mm was performed. These testing parameters were conducted, including the temperature range of 22–200 °C with a ramping of 5 °C·min^−1^, and the frequency of the oscillation at 1 Hz. The storage modulus (*E′*), loss modulus (*E″*) and mechanical loss factor (tan(δ)) were recorded and plotted as a function of temperature.

## 3. Results and Discussion

### 3.1. Fiber Characterization

**Figure 1:**
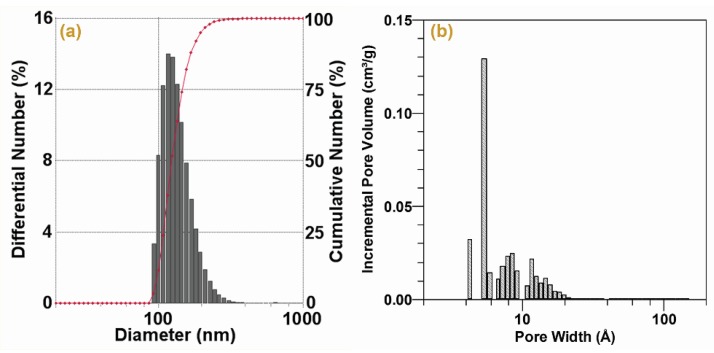
Particle-size distribution (**a**) and DFT pore-size distribution; (**b**) of PAC.

The particle-size and distribution of PAC were determined by the light scattering method. Figure 1a shows that the diameter of the PAC particle was mainly in 95–400 nm with a peak at 140.3 ± 35.6 nm. The average particle size of PAC was calculated to be 265.9 ± 5.1 nm. The specific surface area of the PAC was 835.7 ± 22.3 m^2^/g according to the BET theory, in which the micropore (<2 nm, as defined by the International Union of Pure and Applied Chemistry (IUPAC)) specific surface area was 708.5 m^2^/g (84.8% of the total surface area). The pore structure of the PAC is shown in Figure 1b by means of a DFT model. The total specific pore volume was 0.362 cm^3^/g, and almost all of the pore volume was in the micropore range (Figure 1b).

**Figure 2:**
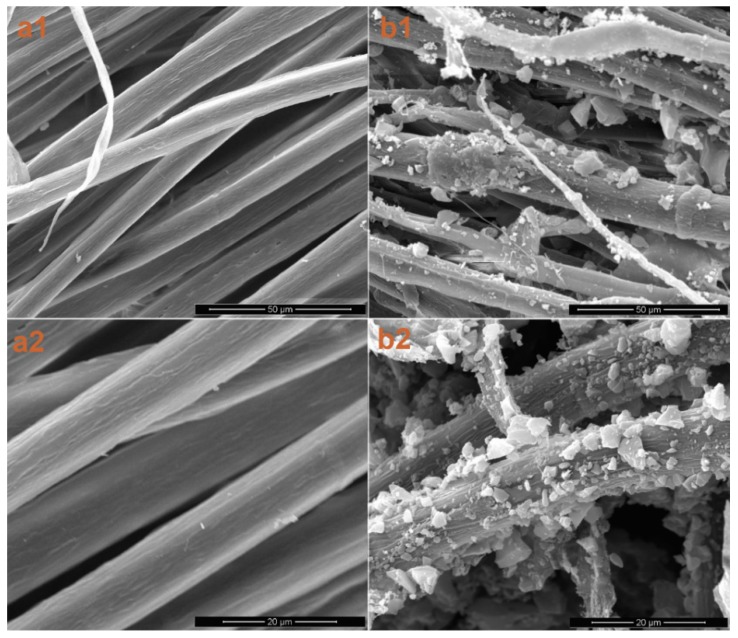
SEM observation of the un-treated fiber (**a1**,**a2**); and Fiber/PAC30 (**b1**,**b2**).

The SEM photos of the un-treated fiber and Fiber/PAC30 are shown in Figure 2 for comparison. PACs on the surface were visible in the Fiber/PAC30 (Figure 2b1,b2), when compared with the un-treated fiber (Figure 2a1,a2). The PAC loading effectiveness was calculated from Equation (3) in terms of BET specific surface area (SA) and the results are shown in Table 1.

(3) $$\text{PAC content }\left(\%\right)=\frac{{\text{SA}}_{\text{Fiber}/\text{PAC}}-{\text{SA}}_{\text{Fiber}}}{{\text{SA}}_{\text{PAC}}-{\text{SA}}_{\text{Fiber}}}\times 100$$

where SA_PAC_, SA_Fiber_, SA_Fiber/PAC10_, SA_Fiber/PAC20_, and SA_Fiber/PAC30_ were 835.6 ± 22.3, 3.3 ± 0.2, 115.1 ± 3.3, 221.0 ± 6.3, and 319.8 ± 9.5 m^2^/g, respectively. The PAC loading effectiveness of each Fiber/PAC was calculated by Equation (4):

(4) $$\text{PAC loading effectiveness }\left(\%\right)=\frac{\text{PAC content}\times \text{Weight of Fiber}/\text{PAC}}{\text{PAC feed}/(1+\text{PAC moisture content})}\times 100$$

where PAC content was calculated by Equation (3), and PAC moisture content was 6.1%. The PAC loading effectiveness was found to be 66.6%, 77.9% and 84.7% for Fiber/PAC10, Fiber/PAC20, and Fiber/PAC30, respectively. These results show that the loading effectiveness increases with the increment of PAC feed. The reason was that hydrophobic PAC was absorbed onto the hydrophilic fiber surface that resulted from the very big specific surface area and high porosity as well as absorbability. The surface of the Fiber/PAC became hydrophobic and absorbed more PAC when the PAC loading further increased, which resulted in more effective absorption for PAC. The results reflect as the increment of the PAC loading effectiveness.

**Table 1:** Contents of PAC loaded fibers.

Specimen	PAC Feed	PAC Loading Effectiveness	Content (%)	
	(g)	(%)	PAC ^a^	Fiber
Fiber	–	–	0.0	100.0
Fiber/PAC10	10	66.6	13.4	86.6
Fiber/PAC20	20	77.9	26.2	73.8
Fiber/PAC30	30	84.7	38.0	62.0

^a^ PAC = powdered activated carbon.

### 3.2. Composites Properties

The physical properties of composites manufactured through VARTM technology are shown in Table 2. The composite density was decreased with the increment in PAC content because of the porosity increment of the composites. The BET specific surface areas of Fiber/polyester, Fiber/PAC10/polyester, Fiber/PAC20/polyester, and Fiber/PAC30/polyester were 0.1, 0.4, 5.0, and 5.5 m^2^/g, respectively, suggesting the increment of the porosity of the composites.

**Table 2:** Contents of the composites.

Composite	Density	Thickness	Content (%)		
	(Kg·m^−3^)	(mm)	Fiber	PAC ^b^	Resin ^c^
Fiber/polyester	1159.4 (44.5) ^a^	2.6 (0.2)	66.7	0.0	33.3
Fiber/PAC10/polyester	1157.3 (15.1)	3.5 (0.1)	64.2	10.0	25.8
Fiber/PAC20/polyester	1064.7 (20.4)	4.2 (0.1)	57.9	20.5	21.7
Fiber/PAC30/polyester	1036.2 (18.8)	4.8 (0.2)	47.1	28.9	24.1

^a^ mean (standard deviation); PAC ^b^ = powdered activated carbon; ^c^ polyester was used here.

**Figure 3:**
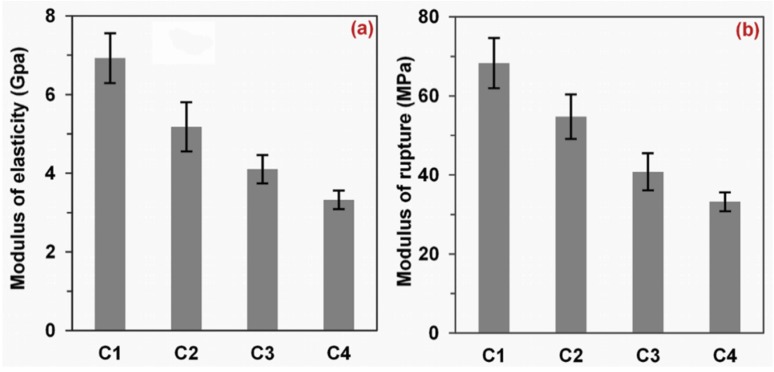
Modulus of elasticity (**a**) and rupture (**b**) of Fiber/polyester (C1), Fiber/PAC10/polyester (C2), Fiber/PAC20/polyester (C3), and Fiber/PAC30/polyester (C4) composites.

From the 3-point bending test results shown in Figure 3, the mechanical properties (MOE and MOR) of the composites decreased with the increase in PAC content, showing the same variation trend as the composite density results. MOE (3.3 GPa) and MOR (33.2 MPa) of the Fiber/PAC30/polyester decreased by 52.0% and 51.4%, as compared with Fiber/polyester (6.9 GPa MOE and 68.3 MPa MOR). The main reason could be that the increment in porosity reduced the mechanical property [26].

**Figure 4:**
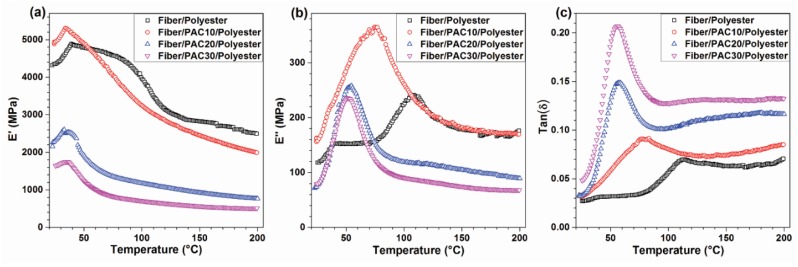
DMA results of the four composites, including storage modulus (**a**); loss modulus (**b**); and damping parameter (**c**).

Storage modulus is a crucial index for measuring the energy storage capability of material after elastic deformation, which was an index of resilience. Figure 4a shows the *E′* of the four composites. In general, the *E′* of the four composites showed the maximum values around 32–39 °C, and then decreased as the temperature increased because of the chain mobility increasing of the polymer matrix [27]. Similar to the mechanical properties (MOE and MOR), after adding PAC, *E′* decreased considerably compared with Fiber/polyester, indicating that additional PAC reduced the elastic properties of the composites. Figure 4b represents the loss modulus of the four composites. The *E″* of Fiber/PAC10/polyester, Fiber/PAC20/polyester, and Fiber/PAC30/polyester composites followed the same trend as that of *E′*. However, the *E″* of Fiber/polyester showed a lower value as compared with that of Fiber/PAC10/polyester composite because of the additional high-porous PAC. Figure 4c shows the loss factor of the four composites, reflecting the damping of molecular movement within the material. As the tan(δ) increased, the flow of the viscous molecules became harder, and the energy increasingly dissipated. With the increase of additional PAC, the tan(δ) increased gradually, indicating that PAC was able to restrict the movement of the chains and thus increase the damping effect. Additionally, as shown in Figure 4c, the glass transition temperatures of the Fiber/polyester, Fiber/PAC10/polyester, Fiber/PAC20/polyester, and Fiber/PAC30/polyester composites were obtained to be 113.4 °C, 83.2 °C, 57.6 °C, and 56.3 °C, respectively, which showed a increment with the amount of added PAC.

### 3.3. EMI Shielding

**Figure 5:**
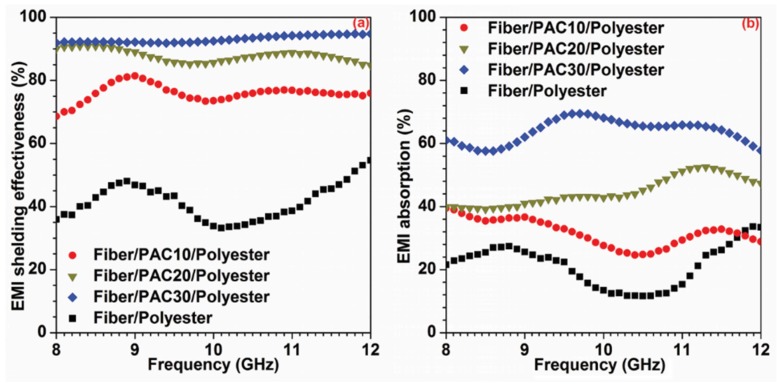
EMI shielding effectiveness (**a**) and absorption (**b**) of the four composites.

Figure 5 shows the EMI shielding and absorption of the four types of composites. The averaged EMI properties at the range of 8–12 GHz are summarized in Table 3. The EMI shielding of Fiber/PAC30/polyester was 93.0%, which was increased by 124.6% compared with the Fiber/polyester composite. Since PAC is different from the common EMI shielding mechanism of reflection, it prefers the absorption of the EMI signals. The EMI absorptions of Fiber/polyester, Fiber/PAC10/polyester, Fiber/PAC20/polyester, and Fiber/PAC30/polyester were 21.2%, 31.7%, 44.7%, and 64.0%, respectively. The EMI absorption of Fiber/PAC30/polyester was increased by 201.7%, as compared with that of Fiber/polyester. The percentages of absorption in the EMI shielding and ratios of EMI absorption/shielding are presented in Table 3. The ratios of EMI absorption/shielding increased from 41.8% to 50.9% and 68.7% for the Fiber/PAC10/polyester to Fiber/PAC20/polyester and Fiber/PAC30/polyester, which indicated that the absorption percentage in EMI shielding increased with the increment of the PAC content. The EMI absorption/shielding of Fiber/PAC30/polyester (68.7%) was significantly increased by 37.1% (ANOVA test, α = 0.001, *p*-value = 9.07 × 10^−79^), compared with that of Fiber/polyester (50.1%), which indicated that the percentage of reflection in EMI shielding was significantly reduced. The increase of absorption resulted in the decrease of the secondary electromagnetic pollution.

**Table 3:** EMI shielding and absorption of the four composites.

Composite	EMI Shielding	EMI Absorption ^a^	EMI Absorption/Shielding
	(%)	(%)	(%)
Fiber/PAC10/polyester	76.0 (2.6) ^b^	31.7 (4.2)	41.8 (5.7)
Fiber/PAC20/polyester	87.9 (1.8)	44.7 (4.4)	50.9 (5.4)
Fiber/PAC30/polyester	93.0 (1.1)	64.0 (3.7)	68.7 (4.1)
Fiber/polyester	41.4 (5.5)	21.2 (6.5)	50.1 (10.3)
Increment (%) ^c^	124.6	201.7	37.1

^a^ EMI = electromagnetic interference; ^b^ mean (standard deviation) which were calculated from the data in 8–12 GHz; ^c^ increments were calculated from the data of Fiber/PAC30/polyester and Fiber/polyester.

## 4. Conclusions

The composites were manufactured from kenaf fibers, PAC and polyester using the VARTM technology. The PAC loading effectiveness was determined using the BET specific surface area analysis, which showed an increase with the increase in PAC loading. EMI shielding tests were carried out with a variable frequency ranging from 8 GHz to 12 GHz. As the PAC content increased from 0% to 28.9%, the EMI shielding and absorption increased from 41.4% to 93.0%, and 21.2% to 64.0%, respectively, and the ratio of EMI absorption/shielding significantly increased by 37.1%. The results showed that PAC was more effective at EMI signal absorption rather than reflection, which benefited the decrease of secondary electromagnetic pollution. However, the mechanical and dynamic mechanical properties decreased after the addition of PAC. Further work is planned to improve these properties of the composites.
